# Intracellular iron accumulation throughout the progression of sepsis influences the phenotype and function of activated macrophages in renal tissue damage

**DOI:** 10.3389/fphys.2025.1430946

**Published:** 2025-01-30

**Authors:** Mira Hanna, Ahmed M. A. Akabawy, Mohamed Mansour Khalifa, Marawan Abd Elbaset, Reda Abdelnasser Imam, Hanan Seddiek

**Affiliations:** ^1^ Department of Medical Physiology, Faculty of Medicine, Kasr Al-Ainy, Cairo University, Cairo, Egypt; ^2^ Department of Biochemistry and Molecular Biology, Faculty of Pharmacy, Helwan University, Cairo, Egypt; ^3^ Department of Medical Physiology, College of Medicine, King Saud University, Riyadh, Saudi Arabia; ^4^ Department of Pharmacology, Medical Research and Clinical Studies Institute, National Research Centre, Cairo, Egypt; ^5^ Department of Anatomy and Embryology, Faculty of Medicine, Kasr Al-Ainy, Cairo University, Cairo, Egypt

**Keywords:** sepsis, acute kidney injury, macrophage polarization, iron homeostasis, macrophage markers, iron transporters

## Abstract

Sepsis, the most common cause of acute kidney injury, remains a major socioeconomic burden. A dysregulated immune response leads to progressive organ dysfunction. Although numerous inflammatory pathways were described, most are still vague and need to be studied in terms of the mechanisms to improve the therapeutic intervention. We tackled the relationship between intracellular iron overload and macrophage polarization within 6, 24, and 72 h of sepsis induction. In our study, sepsis-induced kidney injury was caused by using the cecal ligation and puncture (CLP) model. Our results indicated severe renal tissue damage with a progressive increase in serum BUN and creatinine with architectural tissue damage and positive PAS staining. There was increased expression of CD8^+^ CD68^+^ M1 macrophage markers with upregulation of iNOS and co-expression of CD163^+^. Alternatively, Arg1^+^ Fizz1^+^ M2 macrophage markers were downregulated with increased iNOS/Arg1 ratio. TFR1, cubilin, and DMT1, as iron transport systems, were increased compared to sham but were significant after 72 h, while ZIP8 showed no significant change. There was a correlation between iron overload and M1 macrophage polarization with CD163^+^ phenotype, together with fibrotic changes. The intracellular iron overload with downregulation of ferritin was strongly related to macrophage polarization that was exaggerated at 72 h. Finally, early introduced therapy to target free iron during sepsis is a proposed novel solution for protecting the renal tissue from acute injury due to macrophage activation that may end up with chronic kidney injury, if not mortality.

## 1 Introduction

Acute kidney injury (AKI) induced by the dysregulated inflammatory response to infection as part of sepsis is one of the most common end-organ dysfunctions. It accounts for 26%–50% of all AKIs, which may lead to severe morbidity and mortality. In addition to being frequent and profound, the underlying pathophysiologic mechanisms of AKI secondary to sepsis are not fully known and investigated (Dicu-Andreescu et al., 2024; Fan et al., 2023; Sun et al., 2023; Zarjou and Agarwal, 2011).

Polarization of macrophages, one of the cells of innate immunity, plays a central role in septic inflammation. In case of AKI due to a septic inflammatory process, macrophages (M0) are activated to M1 (iron-retaining cells), which has pro-inflammatory potentials, while M2 (iron-releasing cells) has a general anti-inflammatory effect with different subtypes (Locati et al., 2020; Kumar, 2019; Alikhan and Ricardo, 2013).

Despite that, iron is an essential element for different cellular physiological functions, including immune balance. However, an overload of labile iron generates free oxygen species, sharing in the oxidative stress that leads to cellular injury. This may play a crucial role in AKI (Scindia et al., 2019). So, iron homeostasis in the body is greatly affected by macrophages because they absorb iron and store it as ferritin. On the other hand, they play a role in the degradation of ferritin and liberate iron in the inflamed tissue and in serum, which may affect the injurious level of the tissue and healing capability (Patino et al., 2023; Recalcati and Cairo, 2021; Scindia et al., 2019; Cronin et al., 2019; Winter et al., 2014).

Regulation of iron homeostasis is a precise, complex process that could be an essential key for the treatment of inflammatory and infectious diseases, especially sepsis. Inflammation is accompanied by hypoferremia through the hepcidin–ferroportin inverse proportion relation that negatively affects extracellular bacterial growth while positively affecting intracellular pathogen growth (Mu et al., 2021).

Some studies have shown that the inflammatory response was enhanced by the depletion of iron (Daglas and Adlard, 2018), while others have shown the reverse scenario in which iron overload was coupled with the pro-inflammatory response (Sharawy et al., 2022; Fan et al., 2014). It seems that the iron status affects the inflammatory responses in case of tissue injury. So, whether decreased free iron or its overload affects the inflammatory process accompanying sepsis and determining the mechanism still needs more investigation.

Xia et al. (2021) concluded in their review that iron may be necessary for M1-like macrophage polarization and negatively regulates M2-like macrophage polarization. In some situations, iron may promote M2-like macrophage polarization. In addition, several factors might be involved in the iron–macrophage association. Interestingly, iron status causes different inflammatory response outcomes from iron. They added that a debate has been running after many *in vitro* and *in vivo* studies have been performed on whether iron supplementation or deprivation could affect macrophage polarization toward M1 or M2 phenotype. This needs more intense investigations as the roles of iron in regulating pro-inflammatory macrophage polarization are not always consistent. The conflicting results of studies suggested that iron might regulate macrophage polarization by affecting different signal pathways based on various models. Mostly studied models were cancers/tumors, atherosclerosis, nonalcoholic fatty liver disease, pancreas, adipose tissue, skin, and wound healing (Xia et al., 2021; Winn et al., 2020).

We aimed to investigate the relation between the impact of iron homeostasis’s impact on innate immunity represented by macrophage activation and regarding transport system related to the effect of septic inflammation along a specific 72-h time course in AKI.

## 2 Material and methods

### 2.1 Experimental animals

Male Wistar albino rats weighing 160–200 g were purchased from the National Research Centre animal house. The animal ethics committee of Cairo University approved all experimental procedures (CU-II-F11-20). All animals were housed at five animals per stainless steel cage in a room at a constant temperature and humidity under a 12-h light/dark cycle with *ad libitum* access to tap water and food. They were randomly subjected to sham operation or cecal ligation and puncture (CLP). Thirty-six animals were divided into two main groups (18 per group): the control sham group and the CLP group. Each group was further divided into three subgroups (six per group) according to the sacrifice time (6 h, 24 h, and 72 h after CLP or sham surgery).

### 2.2 Sepsis induction using cecal ligation and puncture (CLP) method

All the rats were anesthetized using isoflurane (1%–3% in oxygen), and then a midline incision 3 cm long was performed. After the exposure of the cecum, the CLP group cecal ligation was performed using a 3.0 silk suture at approximately 1 cm below the ileocecal valve, followed by three times cecal perforation using a 16-gage needle. Then, a small amount of feces was squeezed out into the peritoneal cavity, and the cecum was returned to the peritoneal cavity. The midline cutaneous incision was closed with a 4.0 silk suture. At the same time, the sham group was not subjected to CLP. Post-operatively, the animals were injected with 1 mL pre-warmed saline subcutaneously, received analgesia, and allowed to wake up (Sharawy et al., 2022; Capcha et al., 2021; Zhang et al., 2018; Salman et al., 2014). By the end of the experiment, animals were anesthetized using ketamine (100 mg/kg) and xylazine (10 mg/kg) ip and sacrificed at 6 h, 24 h, and 3 days after CLP or sham surgery by cervical dislocation.

### 2.3 Collection of blood samples and rat tissues

Blood was drawn retro-orbitally using a capillary tube. Sera were separated and stored at –80°C until used. After sacrifice, the kidneys were isolated, weighed, and then either preserved in 10% saline-buffered formalin for subsequent histopathological and immunohistochemical studies or kept at −80°C for biochemical analysis in which the tissue was lysed and homogenized using RIPA buffer (Sigma-Aldrich; R0278). In addition, the kidney tissue was homogenized and prepared for molecular analyses.

### 2.4 Kidney function assessment using serum creatinine (Cr) and blood urea nitrogen (BUN)

Serum concentrations of creatinine (Cr) and blood urea nitrogen (BUN) were evaluated as indicators of renal injury and function using the Creatinine – Jaffè Spectrum MDSS GmbH kit (Cat no: 234001) and Invitrogen™ Urea Nitrogen Colorimetric Detection Kit (Catalog no: EIABUN), respectively, following the manufacturer’s methods.

### 2.5 Assessment of ferritin and transferrin

The protein contents of the homogenates and sera were assayed using the Thermo Scientific™ Pierce BCA high-precision Protein Assay Kit (Catalog no: 23227) according to the manufacturer’s protocol (Pfeiffer and Looker, 2017). The Sandwich ELISA technique was adopted using the Invitrogen^TM^ Rat Ferritin Kit (Cat no: EEL129) and Abcam TM Rat Transferrin Kit (ab137993). Antibodies specific for transferrin or ferritin were pre-coated onto microplate wells where standards and samples were pipetted. Biotinylated secondary antibody was then added, followed by plate washing with a wash buffer. The streptavidin–peroxidase conjugate was added, and the unbound conjugates were then washed away. Finally, after the addition of an acidic stop solution, the absorbance at 450 nm was measured using a microplate reader, and the intensity of the color was proportional to the amount of ferritin and transferrin bound to the immobilized (capture) antibody.

### 2.6 Serum and tissue iron parameter analysis

Abcam TM Total Iron-Binding Capacity (TIBC) and Serum Iron Assay Kit (ab239715) were used to indicate the requisite iron for transferrin saturation and serum iron, respectively. First, all standards, controls, and samples were prepared. Then, in a 96-well flat bottom microplate, and for TIBC assay, the working iron solution was added, followed by TIBC detector, TIBC assay buffer, and TIBC developer solution/TIBC developer. For the serum iron assay, a TIBC assay buffer was used, followed by a TIBC detector and TIBC developer solution/TIBC developer. In-between, 37°C-incubation periods were performed throughout the assays. Finally, for standards and samples, the OD measured as 570 nm at the end of the final incubation was used in calculations. Data analysis and calculations were conducted as described in the attached manual.

### 2.7 Quantitative real-time PCR analysis for macrophage polarization and iron transporters

Following the manufacturer’s protocol, the Direct-zol RNA Miniprep Plus Kit (Cat no: R2072, Zymo Research Corp., United States) was used for total RNA extraction from isolated kidney tissues. A NanoDrop One^TM^ microvolume spectrophotometer (Thermo Fisher Scientific) was used to assess the quantity and quality of extracted RNA. A SuperScript IV One-Step RT-PCR kit (Cat. No: 12594100, Thermo Fisher Scientific, Waltham, MA, United States) was utilized for reverse transcription of extracted RNA, followed by PCR. A 96-well plate StepOne instrument (Applied Biosystems, United States) was used in a thermal profile as follows: 10 min at 45°C for reverse transcription, 2 min at 98°C for RT inactivation and initial denaturation by 40 cycles of 10 s at 98°C, and 10 s at 55°C and 30 s at 72°C for the amplification step. After the RT-PCR run, data were expressed in the cycle threshold (Ct) for the target genes and housekeeping genes. Duplicate analysis was adopted. Normalization for variation in the expression of target genes [(Official Symbol; GeneGlobe ID Cat. No): nitric oxide synthase 2, inducible (Nos2; QT00178325), arginase 1 enzyme (Arg1; QT00177611), resistin-like alpha 1 (Fizz1) (Retnla; QT00181965), solute carrier family 11 (proton-coupled divalent metal ion transporter member 1) (DMT1) (Slc11a1; QT02476873), solute carrier family 39 (zinc transporter member 8) (ZIP8) (SLC39A8; QT02464924), transferrin receptor (Tfrc; QT00416892), and intrinsic factor-cobalamin receptor (Cubilin) (Cubn; QT00174559)] was performed referring to the mean critical threshold (CT) expression values of the glyceraldehyde-3-phosphate dehydrogenase (GAPDH) (Gapd; QT00199633) housekeeping gene by the ΔΔCt method. The relative quantitation (RQ) of each target gene is quantified using the 2^−ΔΔCT^ method. All primers used throughout the experiment were ready-to-use QuantiTect^®^ Primer Assay kits purchased from QIAGEN.

### 2.8 Histopathological and immunohistochemical examination

After the euthanasia of animals, kidney specimens were obtained and embedded in 10% saline formalin and then processed for paraffin blocks. Renal sections underwent staining with hematoxylin and eosin stain (HE) and periodic-acid Schiff (PAS) stains. The PAS stain is the most commonly used to demonstrate the thickness of the glomerular basement membrane in the assessment of renal disease. For immunohistochemistry, renal sections were deparaffinized, antigen-retrieved, H_2_O_2_-blocked, and incubated with 1ry antibodies CD8 (dilution, 1:200), CD68 (dilution, 1:200) and CD163 (dilution, 1:200). Imaging was performed via a Leica camera attached to the microscope. The number of +ve immune-stained macrophages was determined manually as absolute cell count per high power field (HPF). ImageJ software assessed the area % of positive Prussian blue and Sirius red. Under ×400 magnification, each section was randomly selected and photographed for five fields. Renal injury was assessed by the following criteria according to PAS staining (Figures 2I, J) and according to H and E (Figure 5A), and the injury pathology score was evaluated as follows: 0, normal; 1, damage involving less than 25% of the area; 2, damage involving 25%–50% of the area; 3, damage involving 50%–75% of the area; and 4, 75%–100% of the area being affected (Zhao et al., 2016; Miyaji et al., 2003).

### 2.9 Statistical analysis

Before proceeding with the statistical analysis, data values were checked for normality using the Shapiro test and for heteroscedasticity with the Brown–Forsythe test. The data were presented as means ± S.E. Data were processed by two-way ANOVA, followed by Šídák’s multiple comparisons test. In contrast, non-parametric values were presented as median ± interquartile range and analyzed by the Kruskal–Wallis test, followed by Dunn’s test; additionally, the exact p-value is offered within the graph. The correlations were performed using either Pearson’s test for parametric values or Spearman’s test for non-parametric ones. SPSS software, version 21, was employed to perform the statistical analysis, while GraphPad Prism software (version 9, United States) was utilized to establish the represented graphs. The significance level was set to p < 0.05 for all statistical tests.

## 3 Results

### 3.1 Sepsis-induced kidney damage and function deterioration throughout 72 h

**FIGURE 1:**
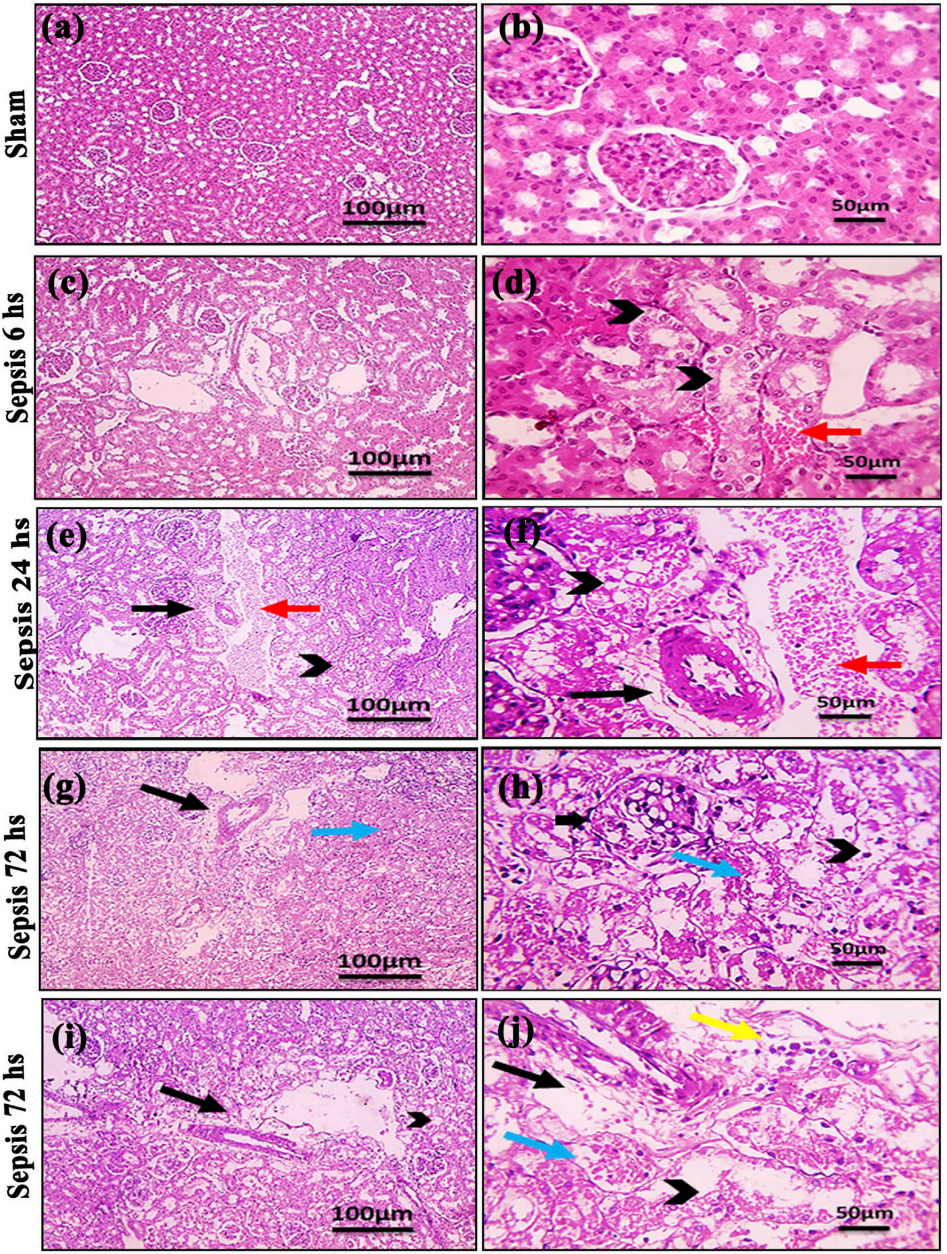
**(A–J)**: Microscopic pictures of HE-stained renal sections showing **(A, B)**: normal glomeruli and tubules in a sham group; **(C, D)**: 6 h after sepsis group showing mild perivascular edema (black arrows), focal areas of starting tubular hydropic degeneration (black arrowheads), and congested blood vessels (red arrow); **(E, F)**: 24 h after sepsis group showing congested blood vessels (red arrows), mild perivascular edema (black arrows), and hydropic degeneration in the tubular epithelium; and **(G–J)**: 72 h after sepsis group showing perivascular edema with infiltration of few leukocytic cells (yellow arrows), severe hydropic degeneration in the tubular epithelium, coagulative necrosis (blue arrows) in the tubular epithelium, glomerular degeneration, and necrosis (thick black arrows). Low magnification X: 100 bar 100 and high magnification X: 400 bar 50.

**FIGURE 2:**
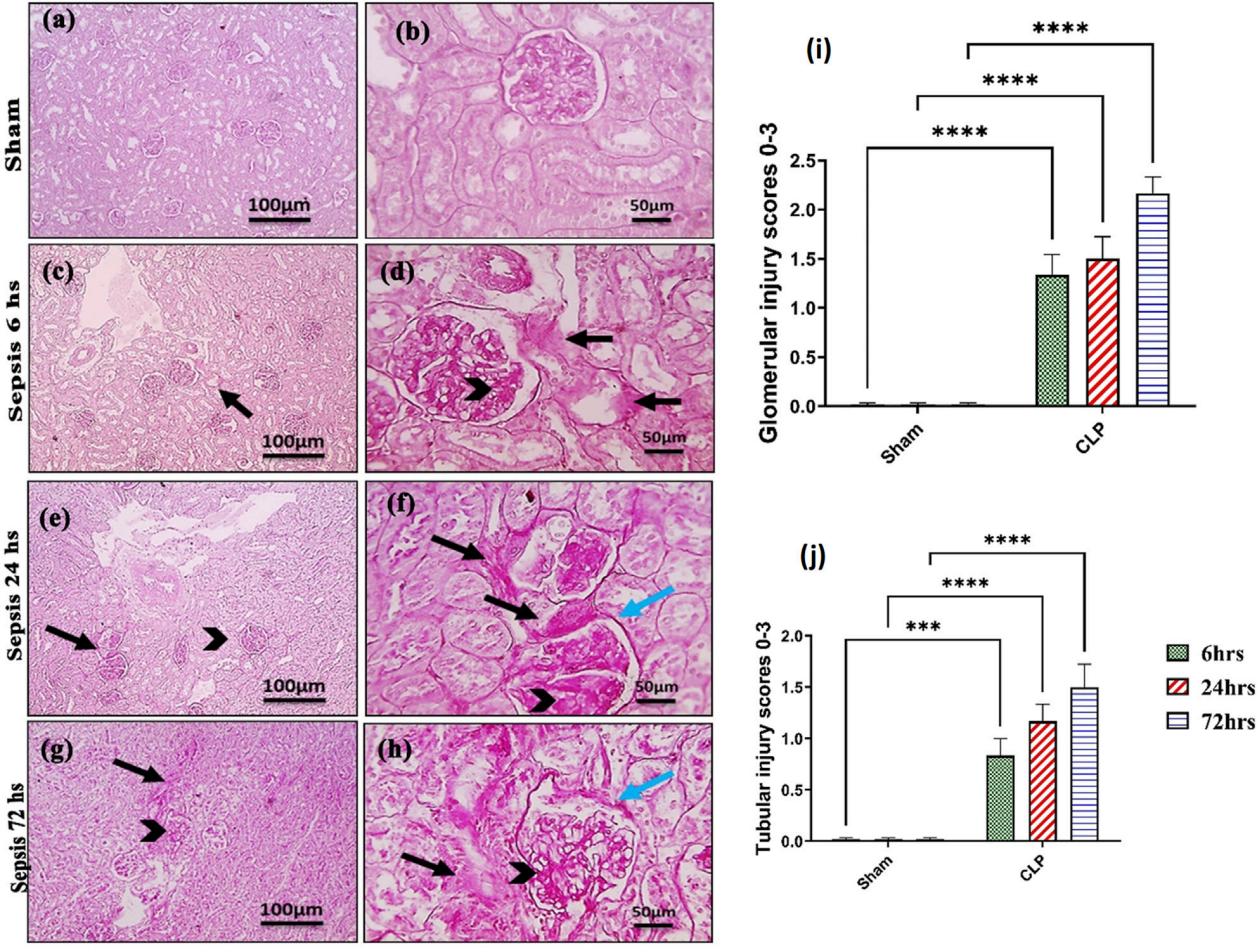
**(A–J)**: Microscopic pictures of PAS-stained renal sections showing **(A, B)**: mild PAS staining (magenta) of glomerular mesangial matrix and basement membranes, minimal PAS staining of tubular basement membranes, and no glomerular sclerosis detected in the sham group; **(C, D)**: 6 h after sepsis group showing enlarged glomeruli, increased PAS staining of glomeruli (black arrowheads) and PAS staining of tubules (black arrows); **(E, F)**: 24 h after sepsis group showing enlarged glomeruli, increased PAS staining of glomeruli with thickened mesangial matrix (black arrowheads) and PAS staining of tubules (black arrows), thickened Bowman’s capsule (blue arrows); **(G, H)**: 72 h after **sepsis** group showing enlarged glomeruli, increased PAS staining of glomeruli with thickened mesangial matrix (black arrowheads) and markedly PAS staining of tubules (black arrows), and thickened irregular Bowman’s capsule (blue arrows). Low magnification X: 100 bar 100 and high magnification X: 400 bar 50; and **(I, J)** statistical figures showing the tubular and glomerular injury score according to PAS staining. All data are expressed as means ± SE (n = 6). Kruskal–Wallis test followed by Dunn’s test. ***means highly significant (P < 0.001) when compared to sham.

**FIGURE 3:**
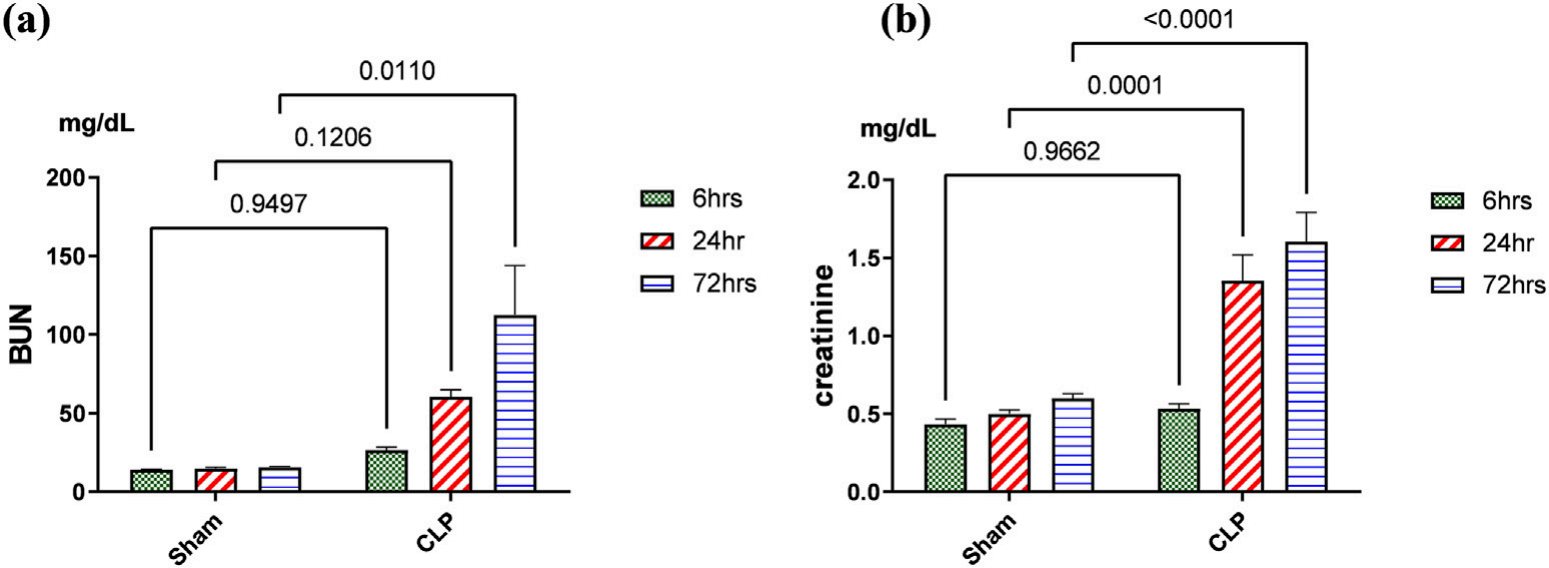
**(A, B):** Kidney functions during 72 h of sepsis-induced kidney injury in which **(A)** shows the significant elevation of serum blood urea nitrogen (BUN) and **(B)** creatinine serum level, especially along the time course of 72 h. Data were expressed as mean ± SE (n = 6). Data were analyzed by two-way ANOVA, and the interaction between time and sepsis was significant for creatinine with a P value of 0.0156. In contrast, the exact p-value of Šídák’s multiple comparisons between sham and disease at respective time intervals is presented within the graph.

Kidney damage, due to inflammatory complications caused by sepsis, was examined by the changes in the histopathologicalstructure of the kidney (Figure 1). It showed progressive deterioration at 6 h after sepsis, renal tissue stained by H & E showed mild perivascular edema with leukocytic cell infiltration, focal areas of starting tubular hydropic degeneration, and congested blood vessels. At 24 h after sepsis, renal tissue showed progressive congested blood vessels, perivascular edema, and well-developed hydropic degeneration in the tubular epithelium. At 72 h after sepsis, renal tissue showed, in addition to congested blood vessels and perivascular edema with leukocytic cell infiltration, severe hydropic degeneration with coagulative necrosis in the tubular epithelium, glomerular degeneration, and necrosis. The kidney injury pathology score detected by histomorphometry revealed a significant gradual increase in the injury score in the CLP group at 6, 24, and 72 h after sepsis compared with sham groups. PAS-stained renal tissue also showed progressive tubular deterioration and glomerular damage compared with the sham group (Figure 2). In addition, 6 h after sepsis, increased tubular staining was observed, while 24 h after sepsis, an increase in tubular and glomerular staining was observed, which were remarkably increased at 72 h after sepsis. PAS density by histomorphometric measurement revealed a significant gradual increase in 6 h after sepsis group, 24 h after sepsis group, and 72 h after sepsis group compared to the control and sham groups. The deteriorated kidney function was confirmed by serum analysis of BUN and creatinine, which showed a significant progressive elevation from 6 to 72 h compared to the sham group (Figure 3).

### 3.2 Changes in the phenotype of activated macrophages accompanied the renal damage

**FIGURE 4:**
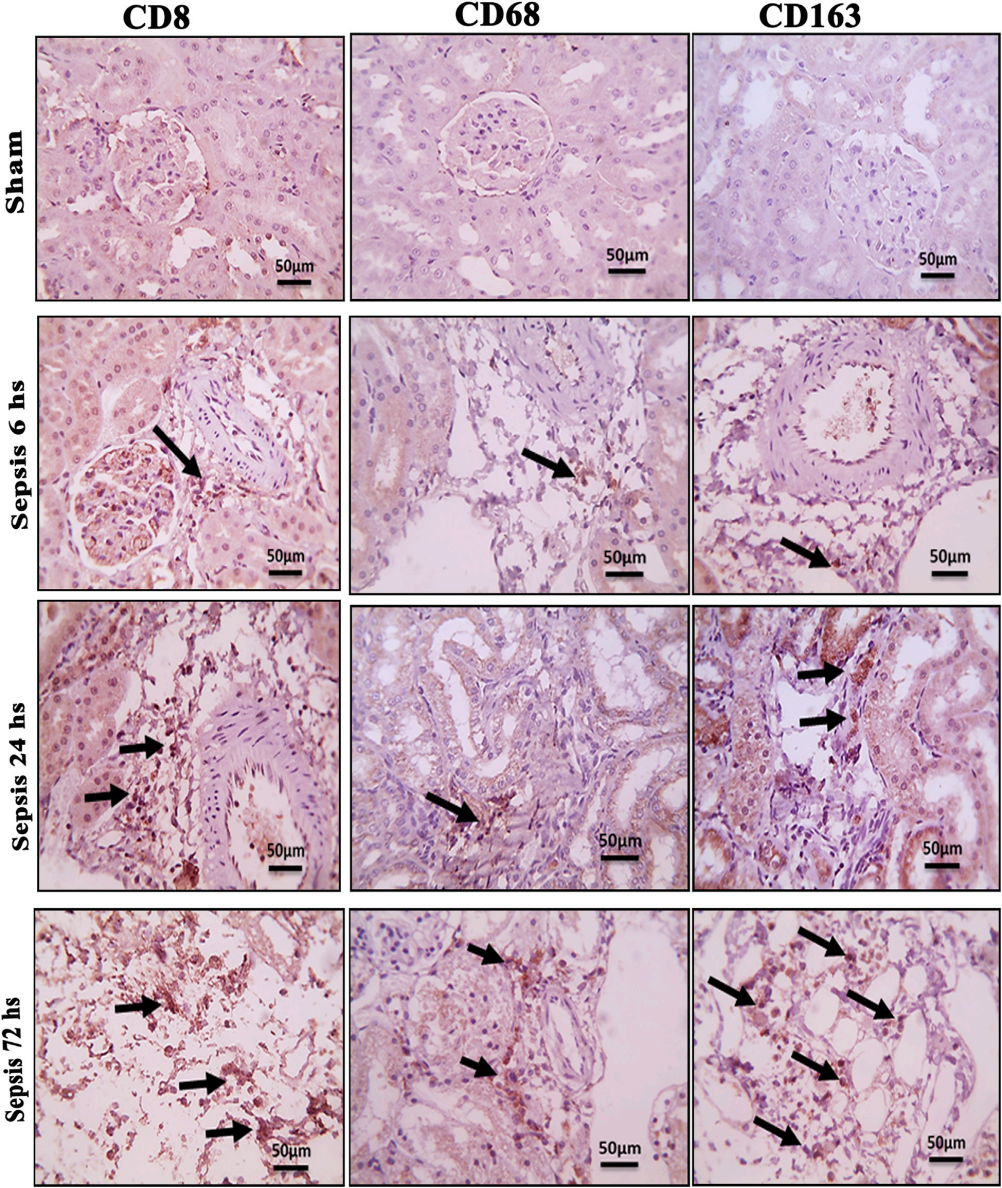
Microscopic pictures of immune-stained renal sections against CD8, CD68, and CD163 show the sham groups with absent positively stained cells; 6 h after sepsis group showed very few positively stained macrophages, mainly located in the perivascular space (black arrows); 24 h after sepsis group showed an increase in the number of positively stained cells; and 72 h after the sepsis group showed a remarkable increase in positively stained cells among the degenerated glomeruli and tubules, particularly those stained with CD8 and CD163. Magnification X: 400 bar 50.

**FIGURE 5:**
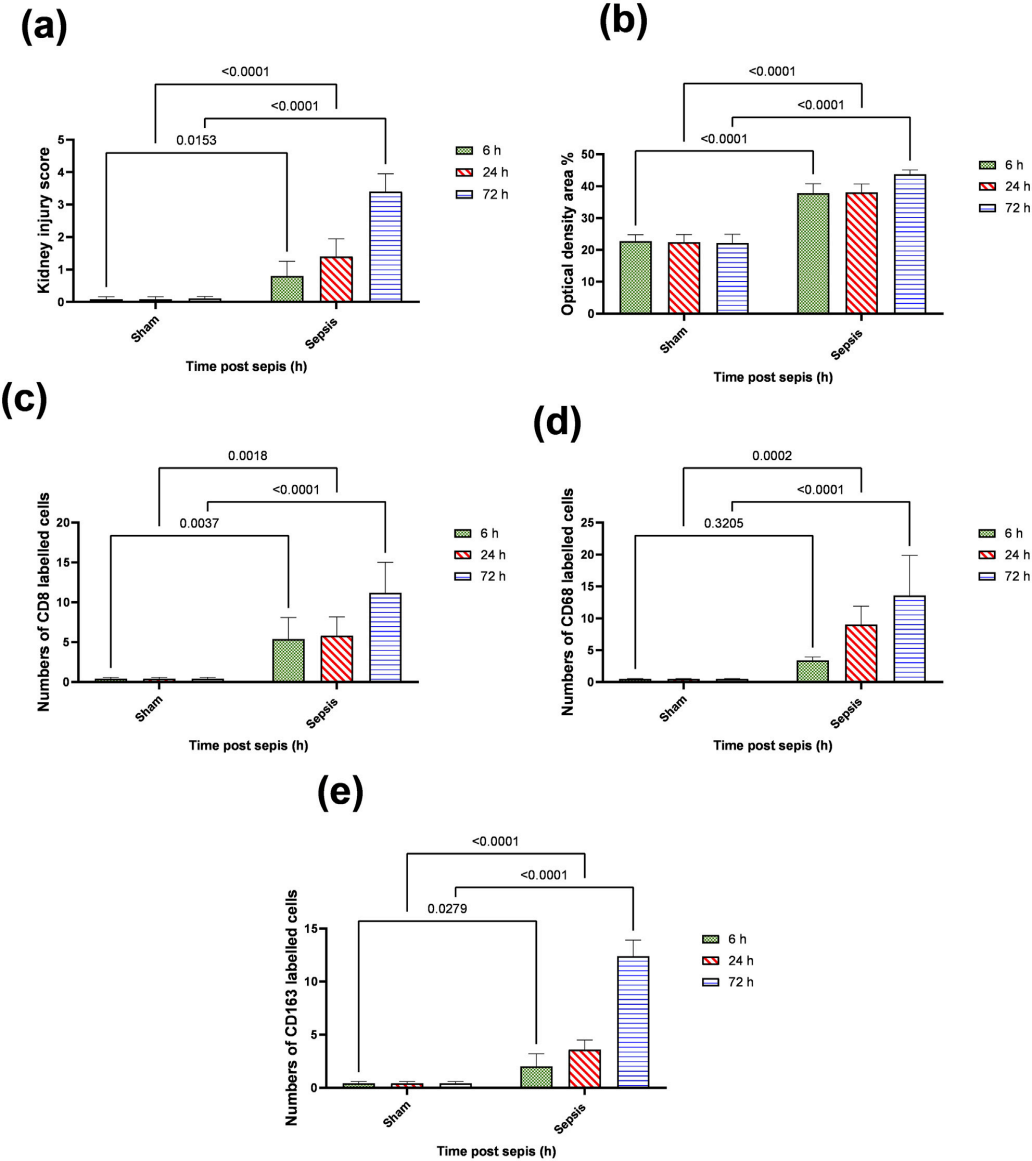
**(A–E)**: statistical figure representing the progressive increase in the expressions of CD8, CD68, and CD163 along the period of the experiment at different time points 6, 24, and 72 h. Data were expressed as median ± interquartile range (n = 6). Data were analyzed using the Kruskal–Wallis test, followed by Dunn’s test. The exact p-value is presented within the graph. Data were analyzed by two-way ANOVA, and the interaction between time and sepsis was significant for CD 68, CD 8, and CD 163, kidney injury score according to H and E, and optical density with P values 0.002, 0.0097, <0.0001, <0.0001, and 0.0095, respectively, while the exact p-value of the Šídák’s multiple comparisons between sham and disease at the respective time interval was presented within the graph.

**FIGURE 6:**
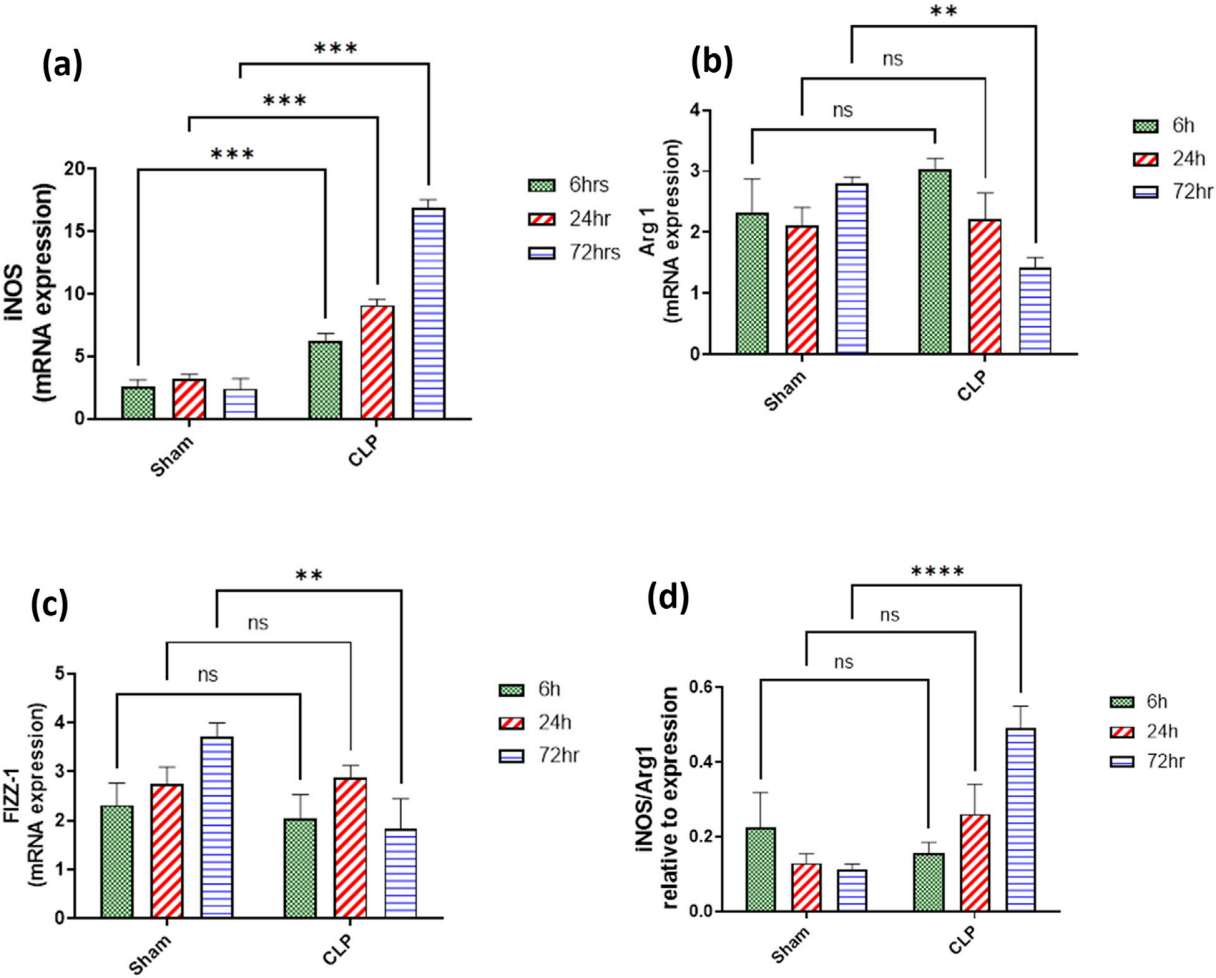
**(A–D)**: RT-PCR mRNA expression levels of macrophage phenotype genetic markers at 6, 24, and 72 h after sepsis-producing acute kidney injury. **(A)** iNOS (Nos2), **(B)** Arg1, **(C)** Fizz1, and **(D)** iNOS/Arg-1 ratio. Expression data are recorded as the mean ± SE (n = 6) of three assays in duplicate normalized to GAPDH and represented as compared with the mRNA expression levels of the corresponding time-interval sham–control group. Data were analyzed by two-way ANOVA, and the interaction between time and sepsis was significant for iNOS, Arg1, and iNOS/Arg1 with P values < 0.0001, 0.0089, and 0.0024, respectively, while the exact p-values of the Šídák’s multiple comparisons between sham and disease at respective time intervals were presented within the graph.

The histopathological structural changes of the renal tissue were accompanied by macrophage polarization. We assessed the activated macrophage types to detect their role in renal damage following sepsis at different time points. The M1 type, which has been known to be pro-inflammatory, was assessed using immunohistochemical analysis of CD68 and CD8, in addition to the genetic expression of iNOS (Figures 4–6A). On the other hand, the M2 type that has been known to be anti-inflammatory and immunosuppressive and tumor-associated macrophages (TAM) as well were assessed using immunohistochemical analysis of CD163, in addition to the genetic expression of Arg1 and Fizz1 (Figures 4–6B, C). A consequence of renal injury due to sepsis recruitment of M1-type macrophages was increased CD8 and CD68 count, which showed a significant progressive increase along the experimental period and was the highest at 72 h. There was a significant increase compared to the nearly negatively stained control and sham groups at the same time points. In addition, there was a significant upregulation of iNOS expression at 6, 24, and 72 h after induction of sepsis.

Regarding M2-type macrophages, Fizz1 and Arg1 showed no significance at 6 and 24 h compared to the sham group, while at 72 h, there was a significant decrease compared to the sham group; however, no significance was detected compared with other time points (6 and 24 h), indicating a minimal decrease at 72 h. At the same time, the expression of CD163 was increased, which was also significantly remarkable at 72 h. On the other hand, for the iNOS/Arg1 ratio, an increase was observed at 6 and 24 h compared to sham, but that increase was only significant at 72 h and significant compared with the sepsis at 6 and 24 h (Figure 6D). This showed that there was a remarkable increase of M1 at the expense of M2. However, CD163^+^ macrophages increased over time with deterioration of renal function and histopathology.

### 3.3 Acute kidney injury due to sepsis is related significantly to the accumulation of iron in renal tissue rather than increased systemic iron through the reduction of iron storage protein ferritin within 72 h

**FIGURE 7:**
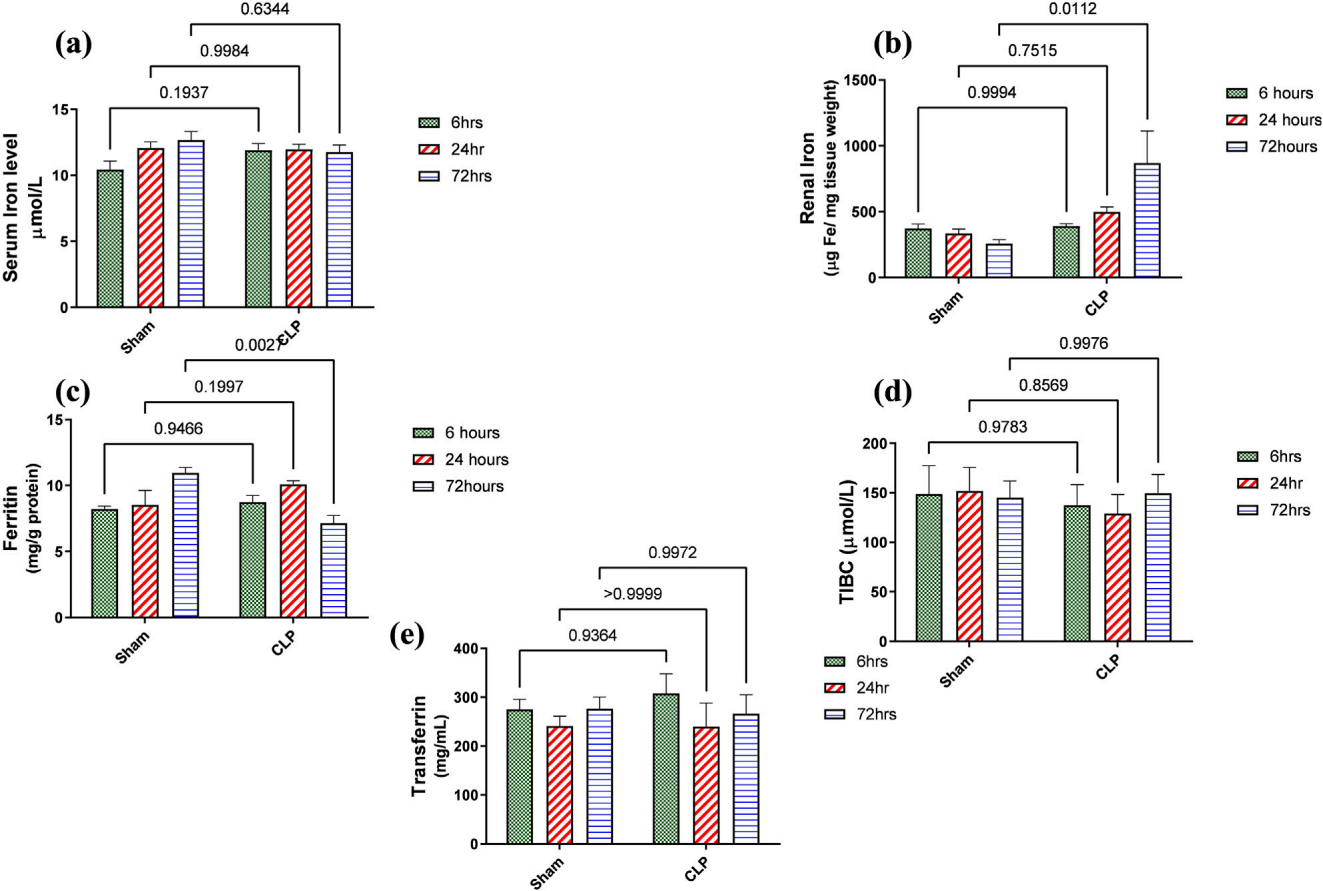
**(A–E)** show the results of iron load assessment in the form of quantitative analysis of **(A)** serum iron level, **(B)** iron accumulation in renal tissue, **(C)** ferritin, **(D)** total iron binding capacity (TIBC), and **(E)** serum transferrin. Data were analyzed by two-way ANOVA, and interaction between time and sepsis was significant for ferritin with a P value of 0.0013. In contrast, the exact p-value of Šídák’s multiple comparisons between sham and disease at respective time intervals was presented within the graph (n = 6).

**FIGURE 8:**
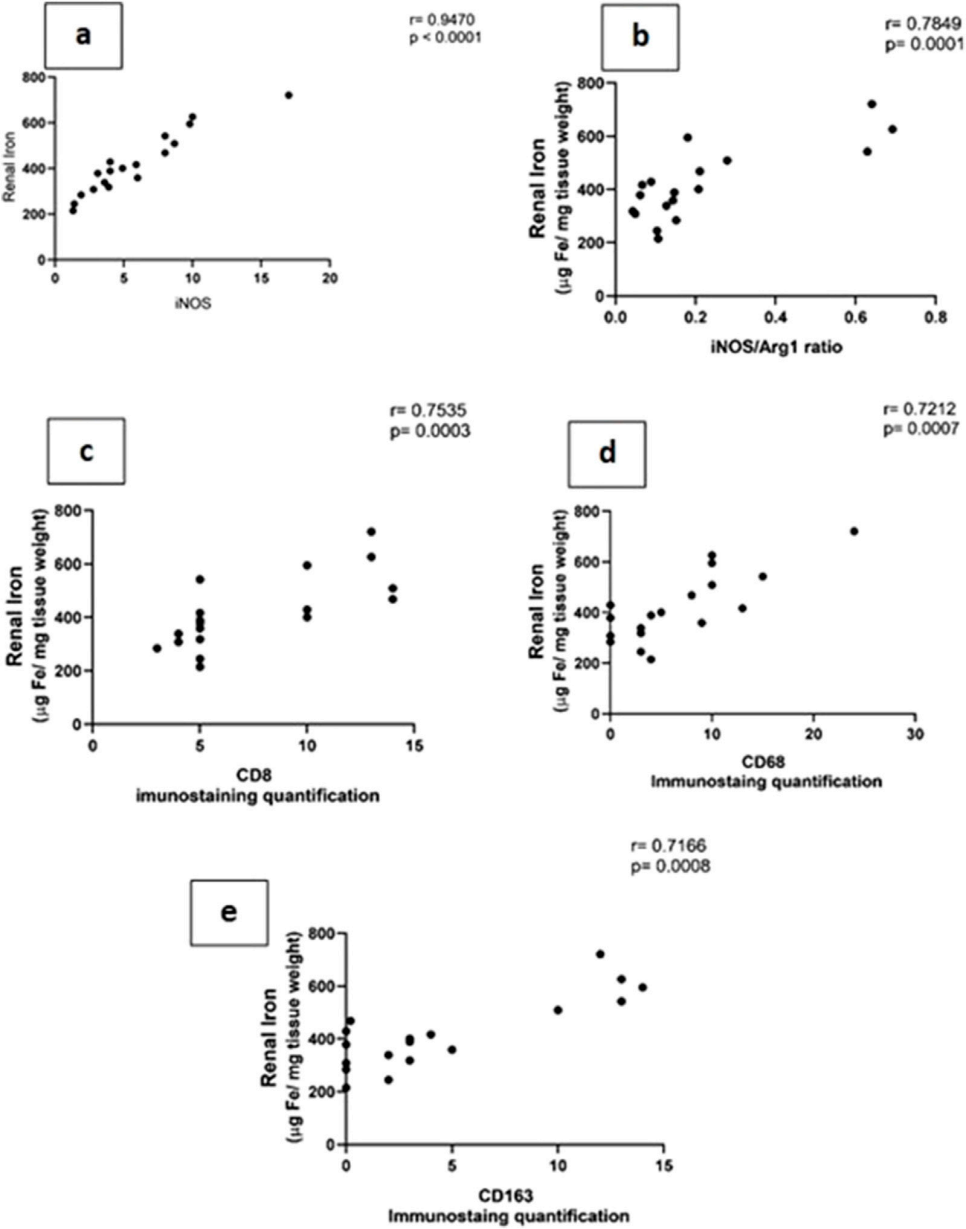
**(A–E)**: show the correlation between renal tissue iron with iNOS (Nos2) **(A)** iNOS/Arg1 ratio **(B)**, CD8^+^ **(C)**, CD68^+^, **(D)** and CD163^+^ **(E)** macrophage phenotypes.

Iron metabolism is affected by macrophage polarization and their differentiation into different types due to their crucial role in iron homeostasis. One of the defense mechanisms is iron sequestration from bacteria, attenuating their proliferation. To assess the iron load systemically and in renal tissue, renal and serum iron, ferritin, total iron binding capacity (TIBC), and serum transferrin were measured. There was an increase in the renal iron concentration, which started at 6 h from the induction of sepsis and was significant at 72 h compared to the sham group. On the other hand, the ferritin content in the renal tissue showed a significant decrease at 72 h. In addition, there was no significant change detected during the experimental period (72 h) in serum iron, TIBC, and serum transferrin between the sham and CLP-induced sepsis groups (Figures 7A–E). There was also a strong correlation between renal tissue iron overload with iNOS upregulation iNOs/Arg1 ratio, CD8^+^, CD68^+^, and CD163+ macrophage phenotype (Figures 8A–E), indicating the impacts of both iron overload and inflammatory macrophages on each other within 72 h of induction of sepsis.

### 3.4 Sepsis-induced changes in iron uptake systems during 72 h in inflammatory nephropathy

**FIGURE 9:**
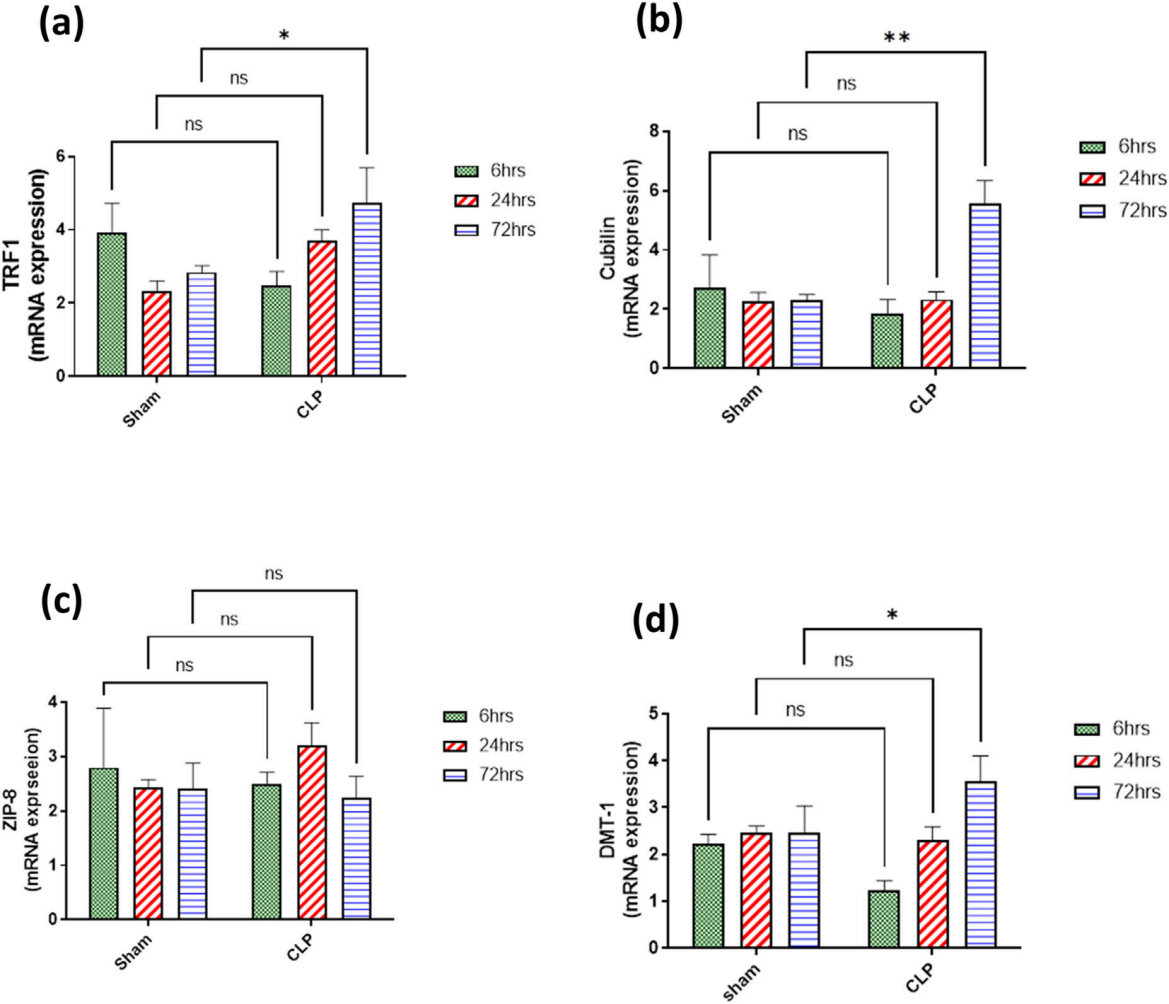
**(A–D)** show the results of iron uptake systems **(A)** TFR1, **(B)** cubilin, **(C)** ZIP8, and **(D)** DMT1. mRNA expression data are recorded as the mean ± SE (n = 6) of three assays in duplicate normalized to GAPDH, as compared with the mRNA levels of the corresponding time-interval sham–control group. Data were analyzed by two-way ANOVA, and the interaction between time and sepsis was significant for TFR1, cubilin, and DMT1 with P values 0.0136, 0.0109, and 0.0265, respectively. In contrast, the exact p-value of Šídák’s multiple comparisons between sham and disease groups at respective time intervals was presented within the graph.

The main iron uptake systems are either transferrin-bound or non-transferrin-bound systems. Macrophages play an important role in the modulation of these systems and hence affect the inflammatory changes in the renal tissue. So, in our study, the transferrin iron-bound system was determined by transferrin receptor protein 1 (TFR-1) and cubilin and non-transferrin iron-bound system by divalent metal transporter 1 (DMT-1) and zinc transporters (ZIPs). There was a significant increase in the expressions of TFR1 and cubilin in the septic renal tissue at 72 h compared to the sham group. DMT1 expression showed a gradual increase that was only significant at 72 h compared to the corresponding sham group. However, ZIP8 showed a non-significant change during 72 h of sepsis (Figures 9A–D).

### 3.5 Sepsis showed iron deposition that increased at 72 h and the start of fibrotic changes at 24 h

**FIGURE 10:**
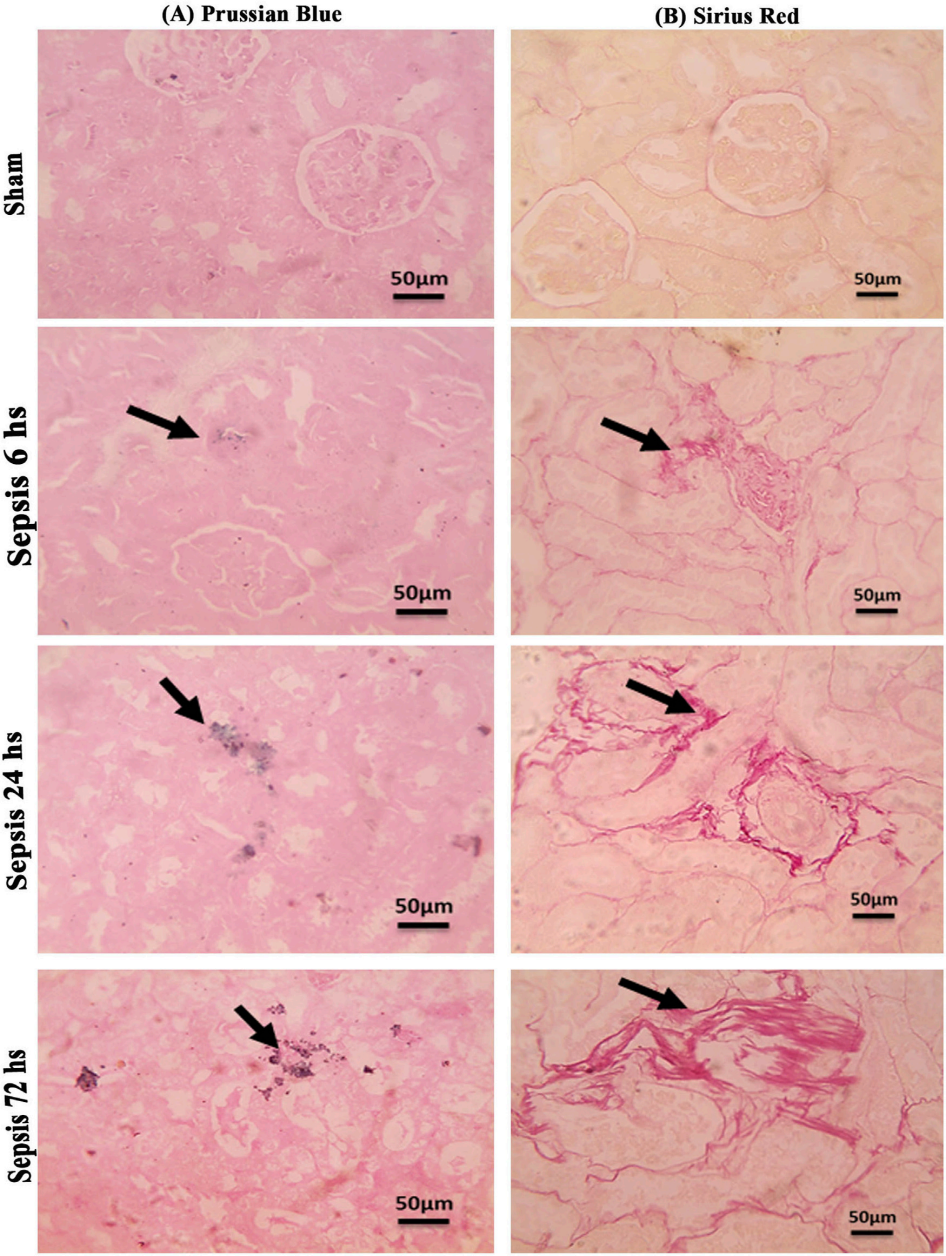
**(A, B)**: **(A)**: Microscopic pictures of Prussian blue-stained renal sections showing no iron deposits in renal tubules in the sham group. Renal sections 6 h after sepsis show mild bluish iron deposits in individual renal tubules (arrow). Renal sections 24 h after sepsis show mild bluish iron deposits in a few renal tubules (arrow). Renal sections 72 h after sepsis show slightly higher bluish iron deposits in a few renal tubules (arrow). **(B)**: Microscopic pictures of Sirius red-stained renal sections showing no fibrosis in the sham groups. Renal sections 6 h after sepsis showing mild interstitial fibrosis (arrow). Renal sections 24 h after sepsis show increased interstitial fibrosis (arrow). Renal sections (arrow) 72 h after sepsis exhibit a marked increase in Sirius red stain—high magnification X: 400 bar 50.

**FIGURE 11:**
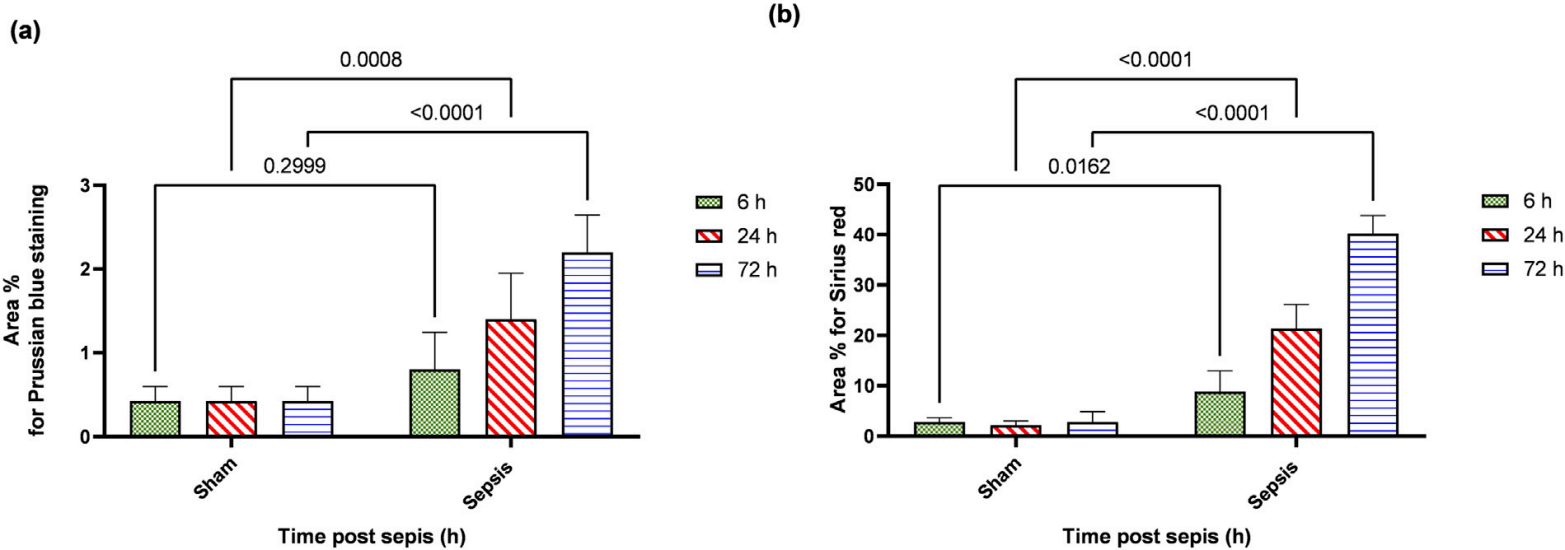
**(A, B)**: show the statistical results of area % of Prussian blue **(A)** and Sirius red.

Prussian blue-stained renal sections showed no iron deposits in renal tubules in the sham group, with mild bluish iron deposits in individual renal tubules in the 6 h after sepsis group. Renal sections 24 h after sepsis show mild bluish iron deposits in a few renal tubules, whereas at 72 h after sepsis, they showed slightly higher bluish iron deposits in a few renal tubules. Sirius red-stained renal sections showed no fibrosis in the sham groups, with mild interstitial fibrosis in the 6 h after sepsis group. Renal sections 24 h after sepsis showed increased interstitial fibrosis, whereas 72 h after sepsis, a marked increase in Sirius red staining was observed (Figures 10A, B; Figures 11A, B).

## 4 Discussion

Sepsis is a significant sequel of tissue injury, especially following infection, leading to end-organ dysfunction due to a severe inflammatory reaction. Acute renal failure following sepsis is one of the major complications that endanger patients’ lives, increasing the mortality rate. Because of the interaction between a lot of factors in the consequences of sepsis, finding an appropriate treatment or prevention of deterioration becomes difficult the drugs targeting the inflammatory released juice are the only offered ones to improve the outcome of sepsis (Fan et al., 2023; Shou et al., 2023; Aslankoc et al., 2022; Tilstam et al., 2021). Precisely studying the mechanism by which sepsis affects the tissue in a harmful manner might help in solving the puzzle for better treatment and preventive measures in the future.

Macrophages, as the major players of innate immunity, are considered to have a prominent role in inflammatory renal injury, especially during sepsis. They are usually activated under unique signals arising from the injured tissue microenvironment, which also affect their functional state. They are highly dynamic, with a diverse spectrum of phenotypes and a wide range of multifaceted roles (Huen and Cantley, 2017; Wynn and Vannella, 2016; Sica and Mantovani, 2012). In addition, during an inflammatory reaction of sepsis, early time-period iron overload in the renal tissue impacts the fate of renal function.

Briefly, in our study, sepsis induced by CLP resulted in deterioration of renal functions and renal tissue damage over 72 h. This was accompanied by increased iron load in renal tissue with decreased ferritin accumulation. These changes were associated with M1 macrophage polarization with increased CD8^+^, CD68^+^ expression, and iNOS upregulation, while down regulation of Arg1^+^ and Fizz1^+^ macrophages (M2) with increased expression of CD163^+^ macrophages. The iron overload occurred with the activation of macrophages toward the M1 phenotype more than Arg1^+^ and Fizz1^+^ macrophages (Ni et al., 2022; Xia et al., 2021; Zhou et al., 2018). This led to renal tissue injury in the form of renal tubular necrosis and glomerular destruction, which consequently could lead to hemolysis and hemoglobin accumulation (Guerrero-Hue et al., 2018; Deuel et al., 2016) that may have led to increased expression of CD163^+^ macrophages. CD163^+^ is a hemoglobin receptor that sequesters the released iron (Mertens et al., 2021; Bulters et al., 2018). So, the increased iron load increased the expression of CD163^+^ macrophages in the trial to protect the renal tissue. Despite that, CD163 was known to characterize M2 anti-inflammatory cells. It might also be expressed by M1 cells. In addition, CD163^+^ macrophages were associated with increased Pas-stained cells, indicating renal tissue deterioration and the onset of fibrosis, which was confirmed by Sirius red staining. The iron load in the kidney may be caused by the degradation of ferritin and/or the filtered hemoglobin and hemolysis that occur at the level of tubular necrosis (Moll et al., 2021; Nakagawa et al., 2021; Guerrero-Hue et al., 2018; Huen and Cantley, 2017; Klessens et al., 2017; Deuel et al., 2016; Olmes et al., 2016).

In a detailed discussion of our results, the renal inflammatory injury was studied by inducing sepsis using the CLP model as it led to a multi-microbial pattern of sepsis simulating peritonitis and septicemia (Fan et al., 2023; Tilstam et al., 2021), which aggravate renal tissue damage including acute tubular injury within 72 h (Shou et al., 2023; Huen and Cantley, 2017; Gomez et al., 2014) and glomerular damage with increased PAS staining. This was accompanied by deteriorated renal functions in the form of increased serum BUN and creatinine (Shou et al., 2023; Aslankoc et al., 2022; Xing et al., 2018).

Iron overload in renal tissue occurred during septic inflammation and contributed to renal damage more than the systemic increase in which the serum transferrin level was not affected. It was detected by increased tissue iron and decreased ferritin, an intracellular iron-storage protein. Ferritin protects cells from the oxidative injurious stress of a free iron load. So, this decrease in renal ferritin levels could explain the excess free iron burden of the renal tissue. The impairment of iron storage results as ferritin principally participates in free iron toxicity. It was also detected that ferritin deficiency in the proximal tubule caused increased tubular damage (Kotla et al., 2022; Bolisetty et al., 2015).

Additionally, both TFR1 and cubilin as transferrin iron binding system (TIBS) together with DMT1 and ZIP8 as non-transferrin iron binding system (NTIBS) showed a gradual increase that was only significant at 72 h compared to the corresponding sham group, except for ZIP 8 that showed no significant increase. TFR1 and cubilin are principal proteins responsible for the cellular uptake of iron via transferrin (Fe^3+^- transferrin) that increases in case of iron overload (Ma et al., 2022; Masaldan et al., 2018). It was detected that TFR1 was highly expressed on the apical surface of proximal tubules in the cortex, the apical membrane of the Bowman’s capsule, and the apical membrane of the collecting duct in the medulla (Zhang et al., 2007). Transferrin as well was endocytosed by the renal proximal tubules via cubilin and megalin receptors as a mechanism of transferrin binding iron for the uptake of iron (Christensen and Birn, 2002). Accumulated evidence implies that free iron could be toxic, sharing in renal tissue damage (Eid et al., 2017; Walker and Agarwal, 2016) because free iron encourages the formation of free radicals and lipid peroxidation (Rochette et al., 2022; Galaris et al., 2019). The detailed mechanisms of imbalance in iron homeostasis are still not precise enough. Intracellular iron homeostasis is regulated by the interplay of iron import controlled by TFR1 and DMT1 and storage by ferritin. DMT1, an iron (Fe^2+^) transporter protein from endosomes to cytoplasm, plays a prominent role in iron reabsorption from the renal tubular lumen as the highest levels of DMT1 messenger RNA were detected in the renal tissue. DMT1 localized at the lysosomal membrane is responsible for the transport of free iron generated from the lysosomal degradation of ferritin, proteins containing iron, and its release in the lumen of the lysosome (Wlazlo et al., 2021; Yanatori and Kishi, 2019; Walker and Agarwal, 2016; Tchernitchko et al., 2002).

The upregulation of the iron transport mechanism in this study was accompanied by increased M1 macrophage polarization with CD8^+^, CD68^+^, and iNOS^+^ while decreased M2 Arg1+ Fizz1+ expression, while CD163+ macrophages showed upregulation. In the renal tissue, macrophages are believed to be responsible for maintaining iron homeostasis by handling and storing it, which could be toxic to other cells (Recalcati and Cairo, 2021; Soares and Hamza, 2016; Ganz, 2012). The iron released by heme catabolism and transported to the kidney is sequestered in macrophages trying to deprive the bacteria of the iron supply for the sake of killing it (Mu et al., 2021; Yanatori and Kishi, 2019). In addition, ferritin deficiency in the proximal tubule after obstructive injury was accompanied by increased macrophage infiltration and pro-inflammatory macrophage activation (Bolisetty et al., 2015). However, macrophages are activated by iron overload toward the pro-inflammatory phenotype in addition to fibrotic induction (Mu et al., 2021). Moreover, Pereira et al. showed that acute iron deprivation could decrease the severity of macrophage-dependent glomerulonephritis in rats. They suggested that iron deprivation could have an anti-inflammatory immuno-metabolic switch in macrophages (Pereira et al., 2019).

Iron driving macrophage polarization or macrophage impact iron load, as principal immune cells responsible for iron homeostasis, differs according to the changes that occur in the macro and microenvironment and the type of the disease (Lahaye et al., 2021; Xia et al., 2021; Winn et al., 2020; Kao et al., 2020; Wilkinson et al., 2019; Costa da Silva et al., 2017; Kroner et al., 2014; Handa et al., 2019 showed that dietary iron overload alters hepatic macrophage polarization status to M1 in liver disease, leading to steatohepatitis and fibrogenesis. They concluded that iron disrupts the balance between M1/M2 macrophage polarization and leads to macrophage-driven inflammation and fibrogenesis in nonalcoholic fatty liver disease.

Some studies showed that iron overload could stimulate the differentiation of macrophages into inflammatory M1 macrophages in many diseases (Yang et al., 2022; Hu et al., 2019; Zhou et al., 2018; Marques et al., 2016; Laskar et al., 2013; Sindrilaru et al., 2011; Costa da Silva et al., 2017 and that iron shaped macrophage polarization toward an M1 pro-inflammatory phenotype in Lewis lung carcinoma and spinal cord injury (Kroner et al. (2014)). On the other hand, the treatment of macrophages with iron-reducing agents, e.g., iron chelators, iron inhibitors, iron-restricted diet, or hepcidin reduction, promoted the differentiation of macrophages into the M2 phenotype (Ma et al., 2022). However, Malhotra et al., 2019 observed a significant decrease in M1 macrophage numbers in hepcidin-deficient mice compared with non-hepcidin-deficient controls. In addition, Agoro et al. (2018) showed that an iron-deficient diet aggravated the inflammatory reaction through M1 macrophages, while an iron-rich diet upregulated the expression of M2 markers in LPS-induced inflammation in liver and peritoneal macrophages. Wilkinson et al., 2019 showed that tissue iron promotes wound repair via M2 macrophage polarization.

Ma et al., 2022 suggested that reactive iron, not total iron stores, has been initiating inflammatory responses. Physiologically, increased iron loading might promote macrophage polarization to the M2 anti-inflammatory reaction. Active iron overload in macrophages, particularly in pathological conditions, may interfere with iron homeostasis, favoring M1 macrophage polarization and aggravating the inflammatory response. Some other studies discussed the effect of the already polarized macrophages on iron homeostasis. M1 shared in iron sequestration as ferritin in part of an inflammatory reaction against infection to deprive the pathogen of iron, preventing its proliferation. At the same time, M2 was concerned with iron release characters (DeRosa and Leftin, 2021; Mu et al., 2021; Sukhbaatar and Weichhart, 2018; Andrianaki et al., 2018; Weiss and Schaible, 2015).

Cui et al. (2022) found that iron-induced oxidative stress interfered with the upregulation of two crucial determinants of macrophage differentiation and function. They suggested that high levels of non-heme iron interfere with macrophage differentiation by inducing mitochondrial oxidative stress. They concluded that the relation between iron overload affecting macrophage differentiation and function is still debatable. Surprisingly, Preng et al. (2022) showed that both iron deprivation and overload attenuated lipopolysaccharide (LPS)-induced inflammation in the *in vitro* study of endotoxin-polarized alveolar macrophages that were challenged with LPS for 6 h. They indicated 48 metabolites that were altered by either or both main effects. Duan et al. (2023), revealed that a well-timed iron replenishment following anti-inflammation treatment plays a determined role in alleviating AKI induced by ischemia–reperfusion. Xia et al. (2021) concluded in their review that iron may be necessary for M1-like macrophage polarization and negatively regulates M2-like macrophage polarization. In some situations, iron may promote M2-like macrophage polarization (Xia et al., 2021; Winn et al., 2020).

In the current study targeting renal tissue and inflammatory septic changes, the iron overload was accompanied by activation of M1 more than M2 with over-expression of CD163. Regarding the M1 activation triggering renal damage (Inoue, 2017), in this study, CD8 and CD68 were upregulated and correlated with increased renal tissue iron. CD68 is a marker for the detection of the degree of macrophage infiltration during renal injury after the CLP procedure at 24 h. M1 macrophages lead to the production of inflammatory mediators and hence play a crucial role in the early generation of AKI (Xing et al., 2018).

iNOS, inducible nitric oxide synthase acting on L-arginine to produce citrulline and toxic levels of nitric oxide, is also one of the biomarkers of M1 activation. It is highly upregulated in our study, indicating high inflammatory endothelial condition with the recruitment of pro-inflammatory macrophages M1, which is one of the criteria for sepsis (Fan et al., 2023; Xing et al., 2018; Zhang et al., 2014; Waddington, 2002).

For the M2 type, our study showed downregulation of Arg1 and Fizz1 phenotypes, the typical inducers of M2 gene expression. Fizz1^+^ is an intracellular protein in inflammatory zone 1. Arg1^+^ metabolizes L-arginine into urea and L-ornithine and is crucial for collagen synthesis (Xing et al., 2018). Fizz1^+^ Arg1^+^ macrophages are considered reparative M2 type that promote tissue fibrosis and dampen T-cell activation, leading to immunosuppression by locally depleting L-arginine. They directly stimulate tubular cell proliferation (Shin et al., 2022; Arlauckas et al., 2018; Bronte and Murray, 2015; Waddington, 2002). Arginase is known to inhibit the activity of nitric oxide synthase and thus could ameliorate the cytotoxic effect of macrophages that was also detected in glomerular *in vitro* culture studies (Waddington et al., 1996; Liew et al., 1991; Hibbs et al., 1987). In addition, our study showed that the ratio of iNOS/Arg1^+^ was in favor of iNOS over arginase, indicating that the inflammatory macrophages (M1) were overruling throughout the timeline of 72 h (Fan et al., 2023; Waddington, 2002).

On the other hand, CD163^+^, an iron scavenger receptor and hemoglobin–haptoglobin complex receptor, and considered an anti-inflammatory wound-healing macrophage M2 marker, was overexpressed in our study. This could have been explained first by the probability of hemolysis due to glomerulonephritis and tubular cell damage as hemoglobin–haptoglobin complexes could be uptaken by macrophages as a renal defensive mechanism through the CD163 receptor, which is trying to protect the kidney from the harmful effect of hemoglobin as a strong oxidant (Cleary et al., 2010; Polfliet et al., 2006; Kristiansen et al., 2001). It is also reported that CD163 was expressed in several kidney diseases related to the accumulation of hemoglobin in renal tissue, such as IgA nephropathy, macroscopic hematuria with AKI, paroxysmal nocturnal hemoglobinuria, and warm antibody hemolytic anemia (Fervenza et al., 2008; Ballarin et al., 2001). Gutiérrez et al. (2012) found that CD163-expressing macrophages were found in areas surrounding tubules filled with RBC, loaded with iron deposits, and filled with erythrocyte casts. CD163^+^ macrophage score was significantly high even in patients with incomplete recovery, suggesting increased number of macrophage subtypes.

Second, CD163^+^ expression is usually associated with fibrosis and could be expressed by M1. Nakagawa et al., (2021) showed that 62%–78% of CD68^+^ M1 macrophages co-expressed CD163 using double immunofluorescence of fibrotic renal tissue of cisplatin-induced renal fibrosis. CD163^+^ M2 macrophages showed a gradual increase at the mid and late stages in addition to minimal representation in control groups.

Third, it has been stated that the plasticity of macrophage polarization is wide enough to be restricted to changes in the gene expression profiles of a few markers. There might be an overlap of some components of M1 and M2 gene expression in the same macrophage in response to more complex activation signals (Chen et al, 2022; Huen and Cantley, 2017). Their polarization could be affected by at least three different suggested mechanisms, including epigenetic and cell survival mechanisms, external stimuli such as pathogens, and tissue environment (cell–cell interaction and cell–molecule interaction) (Parisi et al., 2018; Shapouri-Moghaddam et al., 2018; Murray, 2017). In addition, it is still controversial whether different types of tissue macrophages are due to the recruitment of differential monocyte populations or due to signals within the microenvironment inducing local differentiation and polarization of macrophages (Auffray et al., 2007; Nahrendorf et al., 2007; Arnold et al., 2007). Palmer et al. (2014) showed that CD163^+^ macrophages predominate over more pro-inflammatory HLA-DR^+^ macrophages and are localized near injured proximal tubular cells in human AKI. They stated that CD163+ M2 macrophages were reported in patients with acute kidney injuries and showed that the majority of the CD68^+^ macrophages were also positive for CD163 in both the patients with acute tubular injury and patients with minimal change disease in which M2 (CD163+) macrophages were found to localize close to the tubular basement membrane of injured proximal tubule cells. Fan et al. also showed a slight increase in M2 markers, concluding that there was an imbalance of macrophage M1/M2 polarization of sepsis (Fan et al., 2023).

Since Arg1 did not increase considerably, especially in relation to iNOS that overrule, so we interpret that CD163 expression increased due to tubular injury that increased hemolysis and led to hemoglobin accumulation and hence iron overload in renal tubules and that induced severe renal injury and thus fibrosis within 72 h, as shown by the increased PAS staining and a Sirius red staining. There was also a correlation between CD163^+^ expression and intracellular iron overload.

Significant collagen deposition indicated by Sirius red staining was observed starting at 6 h and increased by 72 h. This result is consistent with increased CD163^+^ macrophages that have been continuously present in areas of active fibrosis with deposition of type I collagen in injured renal tissue (Ikezumi et al., 2015; Ikezumi et al., 2011). Other studies showed the depletion of the M2 macrophages was protective against progressive interstitial collagen deposition, and their depletion from the fourth day of unilateral ureteral obstruction decreased renal fibrosis (Kim et al., 2023; Meng et al., 2022; Shen et al., 2014). We concluded that the early increase of CD163^+^ induced by iron overload during sepsis-induced collagen deposition would progress the AKI to chronic kidney disease within this timeline. In addition, the M1 activation pro-inflammatory cytokine could induce the activation and trans-differentiation to myofibroblasts (Meng et al., 2022; Tang et al., 2020; Wang et al., 2016). This emphasizes our suggestion that the early start of treatment with iron chelators might have an impact on the prognosis of the diseases. There is definitely a lot to be studied regarding this mechanism from different aspects.

In conclusion, sepsis is considered the first lethal cause leading to AKI with induction of renal failure. Sepsis diagnosis is often delayed despite intensive care unit monitoring. When to start treatment is an important question that needs more precise investigation in pathophysiology in relation to the period. In addition, sepsis is an excellent condition for studying macrophage differentiation and its relationship with intracellular iron overload. Additionally, inflammatory signaling and associated innate immune response are involved in the pathophysiological changes in renal tissues within a few hours after injury due to the recruitment of macrophages in the renal tissue, whether they migrate from the bone marrow to target tissues or differentiate into resident macrophages. Iron overload sculpts the feature of sepsis pathogenesis affecting macrophage polarization. M1 macrophages exhibit pro-inflammatory activities that predominate and are related to the accumulation of intracellular iron, inducing a vicious circle. Macrophages in the renal tissue were CD8^+^ CD68^+^ CD163^+^ with an increased iNOS/Arg1 ratio, which was correlated to increased intracellular iron load.

Moreover, transferrin iron transporters were overexpressed. The expression increased within 72 h of sepsis induction, which indicates the importance of early intervention that should be based on decreasing iron overload and driving macrophage polarization to M2 type with Arg1+ and Fizz1+ phenotypes. That may control CD163+ expression, which may induce renal fibrosis with irreversible renal damage. So, iron overload in the renal tissue during the first 72 h affected macrophage polarization that shared the pathophysiology of acute renal tissue injury up to the start of fibrosis. Early therapeutic intervention within the first 6 h targeting intracellular free iron might prevent AKI that may turn out to be chronic.

## Data Availability

The datasets presented in this study can be found in online repositories. The names of the repository/repositories and accession number(s) can be found in the article/supplementary material.

## Abbreviations

AKI, acute kidney injury; Arg1, arginase-1 enzyme; CLP, cecal ligation and puncture; DMT1, divalent metal transporter 1; Fizz1, intracellular protein found in the inflammatory zone 1; iNOS, induced nitric oxide synthase; TFR1, transferrin receptor 1
